# A New Twist in the Abdominal Aortic Aneurysm Story

**DOI:** 10.1055/s-0041-1729917

**Published:** 2021-10-29

**Authors:** Martin David Tilson

**Affiliations:** 1 Department of Surgery, St. Luke's/Roosevelt Hospital Center, College of Physicians and Surgeons, Columbia University Irving Medical Center, New York, New York

Forty years ago, I published a paper that challenged the dogma that the abdominal aortic aneurysm (AAA) was caused by atherosclerosis. This conclusion was based on a comparison of clinical features of 50 patients with AAA and 50 patients with arterial occlusive disease (AOD). An unexpected finding was that men with AAA outnumbered women by a ratio of 7:1, while there were more women than men in the AOD group. 1

This remarkable discrepancy in incidence by sex had several possible explanations. First, we considered that the AAA gene might be sex linked on the X chromosome. There was precedent for this in the spontaneously aneurysm-prone blotchy mouse, which has a point mutation in the *TIMP-1* gene on the X chromosome. Only the affected males get aneurysms. 2

So, we sequenced the *TIMP-1* gene in a human subject, and we found a single-nucleotide polymorphism (SNP). 3 This finding was confirmed by others, but it turned out to be trivial because the SNP was in the third position of the codon. Until recently, there has been no convincing explanation for male predominance.

In 2019, Tang et al 4 reported that there is significant loss of the Y chromosome (LOY) in male patients with AAA. Although LOY occurs in males during aging, the degree of LOY in AAA patients was greater than expected by comparison to age-matched normal and AOD controls.

If LOY is important in males, the same might explain the occurrence of AAA in Turner syndrome (XO). It is interesting that there is a growing list of LOY-associated diseases. Several of these involve inflammation and autoimmunity. Examples include Hashimoto's thyroiditis 5 and rheumatoid arthritis. 6 For a comprehensive discussion, see a recent paper by Guo et al. 7

Our laboratory had also described, in the early 1990's, roles for inflammation in AAA, 8 as well as autoimmunity, 9 and destruction of aortic integrity by matrix metalloproteinases. 10 Investigation of these aspects of AAA pathobiology has led to the publication of approximately 19,000 scientific papers, according to recent searches of Google Scholar.

LOY has also been associated with the occurrence of numerous cancers and disorders of blood and bone marrow. We noticed many years ago that malignancies were not unusual in our AAA patients. The State of Connecticut had an excellent tumor registry, so one of our coinvestigators undertook the effort to search our lists of AAA and AOD patients. A difference in favor of an association of AAA with malignancy was observed. 11 However, the numbers were small, and the statistical significance was unimpressive ( *p* < 0.05). The finding was not confirmed initially but the issue should be revisited. 12

Finally, the present situation allows me to reflect on a philosophical chestnut. In 2006, a New York Academy of Sciences Symposium on the AAA was organized by myself, Helena Kuivaniemi, and Gilbert R. Upchurch, Jr. The last two talks of the meeting were given by Dr. Kuivaniemi and me. We represented the two alternative approaches to discover the AAA susceptibility gene (or genes). Dr. Kuivaniemi discussed genome wide screening (GWS), 13 and I spoke for the candidate gene approach (CGA). 14 The major advantage of GWS is that it is highly objective because it proceeds with zero assumptions about where in the genome to go looking. I call it the “brute force” approach. The disadvantage is that it is highly resource intensive, requires a great many patients, and involves many cooperating investigators and institutions, not to mention that it is expensive. On the other hand, the CGA can be undertaken by one or a few investigators with a small laboratory and limited resources. All that is needed is an in-depth knowledge of the pathobiology of the disease. It is David versus Goliath. In the case of AAA, it appears that the little guys may have won this time, pending confirmations.
